# Importance and utilization frequency of essential competencies of Korean physical therapists

**DOI:** 10.3352/jeehp.2020.17.24

**Published:** 2020-09-01

**Authors:** Junghyun Choi, Taeyoung Oh, Jae Seop Oh, Wootaek Lim, Jeonhyeong Lee, Seul Ki Han, Yun Sang Park, Hyeok Gyu Kwon, Chang Sik Ahn

**Affiliations:** 1 Department of Physical Therapy, Namseoul University, Cheonan, Korea; 2 Department of Physical Therapy, College of Health and Welfare, Silla University, Busan, Korea; 3 Depatment of Physical Therapy, College of Healthcare Medical Science and Engineering, Inje University, Gimhae, Korea; 4 Department of Physical Therapy, College of Health and Welfare, Woosong University, Daejeon, Korea; 5 Department of Physical Therapy, Daegu Health College, Daegu, Korea; 6 Department of Physical Therapy, Daejeon Health Institute of Technology, Daejeon, Korea; 7 Rehabilitation Medicine, Seoul National University Hospital, Seoul, Korea; 8 Department of Physical Therapy, College of Health Science, Eulji University, Seongnam, Korea; Hallym University, Korea

## Background/rationale

It is difficult to present a single definition of physical therapy because physical therapy is defined in different ways in various countries, depending on the length of education required and legal circumstances. However, the World Federation of Physical Therapists defines physical therapists as “persons engaged in healthcare related to providing functional enhancement, damage prevention, rehabilitation treatment, intervention, and recovery service, while maintaining and developing motion and functional capabilities when individuals’ movements and functional capabilities are impaired by age, damage, disease, disability, environmental factors, etc.” [1]. The Korean Physical Therapists Association defines physical therapy as “helping to relieve patients’ pain and further restoring normal social activities by developing and applying physical materials, such as electricity, light, water, air, sound, and exercise, and various instruments and machines for therapeutic purposes, rather than surgery and pharmacological therapy” [2]. Upon comparing the 2 definitions, the Korean Physical Therapists Association suggested that physical therapy is performed using physical materials such as exercise therapy and various instruments and machines, whereas the World Federation of Physical Therapists refers to it as encompassing various services, as well as physical materials, and as including information related to diagnosis, evaluation, and prevention. Thus, it is necessary to redefine the competencies of Korean physical therapists. Changes in the population and disease structure, changes in the medical environment (e.g., advances in medical technology), and subsequent changes in medical personnel–related policies are major factors related to the physical therapist competencies required by society [3]. Korea has become an aging society, with >14% of the population aged ≥65 years, and is expected to become a super-aging society in the near future [4]. In preparation for the upcoming super-aging society, the role of physical therapists in disease prevention and the provision of healthcare for the elderly is expected to increase. Furthermore, it is estimated that the national costs of healthcare will continue to increase according to the public’s perception of quality of life, the increase in the use of medical services, the overuse of high-tech expensive medical equipment, and the need for healthcare for the elderly and high-quality medical services. Therefore, it is necessary to improve the quality of physical therapy services to align with changes in the public’s awareness of health promotion and increasing national medical costs [3].

## Objectives

To cope with the changing healthcare environment, it is necessary to investigate the essential competencies in the clinical practice of physical therapists and to reflect these results in the physical therapy training process. Therefore, in this study, we aimed to provide basic data for the training of physical therapists and policy development by analyzing perceptions of the importance of physical therapists’ competencies and the frequency of the utilization of those competencies.

## Ethics statement

Informed consent was obtained from all participants.

## Study design

This was a survey-based descriptive study.

## Participants

We surveyed licensed physical therapists in Korea who had clinical experience in physical therapy using a Google questionnaire on mobile devices and PCs. A total of 296 (99.0%) of the 299 subjects responded to the survey questionnaires from September 16, 2019 to September 30, 2019 (Dataset 1). The characteristics of participants are shown in Table 1.

## Technical information

The survey items consisted of 4 areas: basic medicine (30 questions), diagnosis and evaluation (38 questions), interventions (43 questions), and other competencies (9 questions) including communication capabilities, professional education and development capabilities, and health personnel’s ethical and interpersonal capabilities (Table 2). The 120 evaluation items are presented in Supplement 1 in Korean and in Supplement 2 in English. In addition, the Cronbach α coefficient was calculated for the reliability of the test in each subcategory, and all reliability coefficients were higher than 0.7 (Table 2). The questionnaire was prepared based on the 2012 job analysis of physical therapists, the 2015 job analysis of physical therapists, learning goals, an analysis of national test linkage, and the national test of physical therapists presented by the Korea Health Personnel Licensing Examination Institute [5,6]. The questionnaire used in the study was validated by 9 experts, including a professor of physical therapy. The subareas of each item were detailed and presented to the subjects (e.g., “Do you think this item is important and has a relatively high clinical utilization?”) The survey responses were a 5-point Likert scale ranging from 1 (not entirely important/very low utilization) to 5 (very important/very high utilization) for the importance and frequency of use of the surveyed competencies.

## Statistical methods

The data were analyzed using SPSS for Windows ver. 15.0 (SPSS Inc., Chicago, IL, USA). Frequency analysis was conducted to identify the general characteristics of the research subjects, and repeated-measures analysis of variance (ANOVA) was performed to analyze the differences in the importance and frequency of utilization of competencies according to the 4 categories (basic medicine, diagnosis and evaluation, interventions, and other competencies essential to physical therapists). To examine the statistical significance of differences in scores according to category, a follow-up test was conducted using the least significant difference (LSD) method. The statistical significance level was set at 0.05.

## Differences in the importance of essential competencies of physical therapists according to category

Repeated-measures ANOVA was performed to analyze the importance of essential competencies of physical therapists. First, differences in the categories of basic medicine, diagnosis and evaluation, interventions, and other competencies showed statistical significance (F=129.33, P<0.01), with the following mean and standard deviation (SD) scores: diagnosis and evaluation (4.45±0.48), interventions (4.42±0.50), other competencies (4.36±0.52), and basic medicine (4.06±0.50 ) (Table 2). The LSD post-test for the statistical significance of differences between each category showed the following significant results: basic medicine versus other competencies (mean difference [M_diff_]=0.30, P<0.01), other competencies versus interventions (M_diff_=0.06, P<0.01), and other competencies versus diagnosis and evaluation (M_diff_=0.08, P<0.01) (Table 3).

## Differences in the frequency of utilization of essential competencies of physical therapists

Repeated-measures ANOVA was performed to analyze the frequency of utilization of essential competencies of physical therapists (Table 4). First, an analysis of differences according to category showed statistical significance (F=42.98, P<0.01), with mean and SD scores as follows: other competencies (4.14±0.58), diagnosis and evaluation (4.14±0.62), interventions (4.08±0.63), and basic medicine (3.89±0.50) (Table 4). The LSD post-test for the statistical significance of differences between each category showed the following significant results: basic medicine versus other competencies (M_diff_=0.30, P<0.01), other competencies versus interventions (M_diff_=0.01, P<0.01), and other competencies versus diagnosis and evaluation (M_diff_=0.03, P<0.01) (Table 4).

## Interpretation and suggestions

Previously, physical therapy in Korea focused on treatment using basic medical techniques using physical materials, such as electricity, light, water, air, sound, and exercise therapy, as well as various instruments and machines, due to the limitations of the medical system wherein interventions are conducted under the guidance of doctors (Table 4). However, it is becoming increasingly important for physical therapists to focus on their diagnosis and evaluation capabilities in order to interpret patients’ status (Table 3). Therefore, for future physical therapy curricula and national exams to present reasonable educational objectives that reflect important practical job competencies of physical therapists, it is necessary to increase the number of hours of education on the subjects of diagnosis, examination and evaluation, and clinical decision-making, and to reflect that emphasis in examinations [3]. The resultant improvements in physical therapists’ diagnostic evaluation competency will increase their ability to accurately identify patients’ problems and to verify the effectiveness of interventions and treatment methods based on those results [7,8].

As the role of Korean physical therapists is based on the International Classification of Functioning, Disability, and Health, which integrates the individual and social models of disability, should be taken to identify the multidimensional effects of disease, social participation, and health status. In addition, appropriate treatment interventions and measures should be presented according to the results of analyses using more diverse approaches. Therefore, it is estimated that the frequency of utilizing competencies in the diagnosis and evaluation category and in the category of other competencies (personal and environmental factors, etc.) was higher than that of the competencies in the category of interventions (Table 3). Among the duties of physical therapists, diagnosis and evaluation-related tasks were considered to be more important and more frequently used than those related to interventions. Therefore, it is necessary to increase the proportion of diagnosis and evaluation in the Korean physical therapist training system to match the frequency of utilization of this competency.

## Conclusion

It is necessary to increase the proportion of credits in university education for diagnosis and evaluation–related competencies, which were recognized to be highly important and frequently utilized, as shown in this study, and to develop evaluation criteria that can enhance physical therapy capabilities by reflecting these considerations in the standards for national examination questions.

## Figures and Tables

**Table 1.** General characteristics of the respondents

Characteristic	Category	Person (%)
Sex	Male	159 (53.7)
	Female	137 (46.3)
Age (yr)	20	147 (49.7)
	30	82 (27.7)
	40	42 (14.2)
	>50	25 (8.4)
Highest level of education	College graduate	197 (22.3)
	Master’s	50 (16.9)
	PhD	49 (16.6)
Career (yr)	<3	106 (22.6)
	3–10	102 (18.9)
	>10	88 (19.6)
Place of employment	Primary medical institution	63 (21.3)
	Secondary medical institution	100 (28.7)
	Tertiary medical institution	60 (20.3)
	University	47 (15.9)
	Others	26 (5.1)
Specialization	Musculoskeletal system	140 (47.3)
	Nervous system	127 (42.9)
	Cardiovascular system	8 (2.7)
	Integumentary system	2 (0.7)
	Others	19 (6.4)

**Table 2.** Essential competency areas and subareas

Areas	Reliability (Cronbach’s α)	Subareas	Reliability (Cronbach’s α)
Basic medicine (30)	0.933	Anatomy (10)	0.874
		Kinematics (4)	0.717
		Physical agent modalities (16)	0.936
Diagnosis and evaluation (38)	0.961	Principle of diagnosis and evaluation (8)	0.901
		Musculoskeletal system examination and evaluation (6)	0.874
		Nervous system examination and evaluation (11)	0.911
		Cardiopulmonary blood relation examination and evaluation (6)	0.939
		Clinical decisions (6)	0.905
Interventions (43)	0.969	Musculoskeletal interventions (8)	0.863
		Neurological interventions (8)	0.890
		Cardiopulmonary blood relations interventions (5)	0.898
		Skin system interventions (2)	0.929
		Physical therapy in the community (2)	0.951
		Physical therapy for children and adolescents(4)	0.952
		Physical therapy in sports (3)	0.960
		Physical therapy for the elderly (4)	0.923
		Physical therapy for women (2)	0.942
		Medical care regulations (5)	0.921
Other competencies (9)	0.914	Areas of communication (4)	0.804
		Professional training and development (2)	0.853
		Medical personnel’s ethical and interpersonal personal capabilities (3)	0.912

Parentheses indicate the number of questions.

**Table 3.** Analysis of the importance of essential competencies of Korean physical therapists

Importance of essential competencies for physical treatment	Mean±standard deviation	Sum of squares	Degree of freedom	Mean squared	F-value	Post-hoc
Basic medicine	4.06±0.50	27.94	2.14	13.09	129.33^**^	1<4<2,3
Diagnosis and evaluation	4.45±0.48					
Interventions	4.42±0.50					
Other competencies	4.36±0.52					

1: basic medicine, 2: diagnosis and evaluation, 3: interventions, 4: other competencies (communication capabilities, professional training and development capabilities, and medical personnel’s ethical and interpersonal capabilities).

** P<0.01.

**Table 4.** Frequency analysis of the utilization of essential competencies of physical therapists in Korea

Frequency of the utilization of essential competencies of physical therapists	Mean±standard deviation	Sum of squares	Degree of freedom	Mean squared	F-value	Post-hoc
Basic medicine	3.89±0.89	13.08	2.32	5.64	42.98^**^	1<3<2,4
Diagnosis and evaluation	4.14±0.14					
Interventions	4.08±0.08					
Other competencies	4.16±0.16					

1: basic medicine, 2: diagnosis and evaluation, 3: interventions, 4: other competencies (communication capabilities, professional training and development capabilities, and medical personnel’s ethical and interpersonal capabilities).

** P<0.01.

## Footnotes

**Data availability** Data files are available from Harvard Dataverses: https://doi.org/10.7910/DVN/XYM7XQ Dataset 1. Response data from 299 subjects.

## Supplementary materials

Data files are available from Harvard Dataverses: https://doi.org/10.7910/DVN/XYM7XQ

Supplement 1. Survey items in Korean.

Supplement 2. Survey items in English.
